# Prognostic impact of anal cancer morphology on survival and local recurrence: A 20‐year regional cohort study

**DOI:** 10.1111/codi.70541

**Published:** 2026-07-05

**Authors:** Alwin Puthiyakunnel Saji, Thomas Rackley, James Horwood, Rachel Hargest

**Affiliations:** ^1^ School of Medicine Cardiff University Cardiff UK; ^2^ Velindre Cancer Centre Cardiff UK; ^3^ Department of Colorectal Surgery University Hospital of Wales Cardiff UK

**Keywords:** anal cancer, overall survival, prognosis, recurrence, tumour morphology

## Abstract

**Aim:**

The relationship between macroscopic tumour morphology and clinical outcomes has previously been demonstrated for malignant melanoma and gastric cancers, but not for anal squamous cell cancer (ASCC). This study investigated the effect of the morphology of ASCC on oncological outcomes.

**Methods:**

The morphology of all ASCC patients presenting between 2003 and 2022 was analysed retrospectively and classified as Exophytic, Ulcerated or Mixed. The primary outcome was 5‐year overall survival (OS). Secondary outcomes were complete response to treatment, local recurrence (LR) and 5‐year disease‐free survival (DFS).

**Results:**

Of 622 patients referred to the anal MDT, 515 were included for analysis: 290 exophytic, 179 ulcerated and 46 mixed morphology. Ulcerated morphology was associated with a significantly lower percentage of patients surviving at 5 years compared to exophytic and mixed groups (25.1% vs. 67.9% vs. 65.2% *p* < 0.001). Ulcerated and mixed morphology patients presented with significantly larger tumours (T4‐ 30.7% vs. 28.3% vs. 10.0% *p* < 0.001) and greater nodal involvement (N2‐ 26.3% vs. 17.2% vs. 15.2% *p* = 0.042). Exophytic morphology patients had significantly greater complete response at 95.9%, compared to 82.6% for mixed and 45.8% for ulcerated tumours (*p* < 0.001). Morphology remained significant in both univariable and multivariable Cox regression models across 5‐year OS, DFS and LR. Ulcerated [HR 5.160 (3.338–7.979) *p* < 0.001] and mixed morphology [HR 2.299 (1.179–4.482) *p* = 0.015] had significantly worse risk of 5‐year overall survival compared to exophytic morphology.

**Conclusion:**

Ulcerated morphology is associated with advanced tumours, poor response to chemoradiotherapy, resulting in a higher incidence of recurrent disease and worse overall‐ and disease‐free survival.

**What does this paper add to the literature?**

Tumour morphology has been correlated to outcomes in various cancers, but not in anal cancer. In this paper, ulcerated morphology is associated with advanced tumours, poor chemoradiotherapy response, higher recurrence rate and worse survival. This is the first study in the literature demonstrating a correlation between anal cancer morphology and oncological outcomes.

## INTRODUCTION

Anal squamous cell carcinoma (ASCC) is a relatively rare condition accounting for 2% of gastrointestinal malignancies [1]. However, its incidence is increasing due to the rise in human papillomavirus (HPV) infections, to which almost all cases of ASCC can be attributed [2, 3, 4]. The natural history of ASCC is thought to be very similar to other HPV‐related cancers, such as cervical cancer [5]. ASCC may initially present with pre‐cancerous anal intraepithelial neoplasia (AIN), which can progress into high‐grade AIN and eventually to ASCC [6, 7]. Multiple studies and meta‐analyses have concluded that the risk of developing ASCC is markedly elevated in patients with certain risk factors, including Human Immunodeficiency Virus (HIV) infection, men who have sex with men (MSM), women with vulval, vaginal or cervical intraepithelial neoplasia (VIN/VaIN/CIN) or cancers and patients who are immunocompromised or taking immunosuppressants [8, 9, 10].

Management of ASCC has changed significantly over the past three decades, moving away from primary surgery [abdomino‐perineal resection (APR)] to primary chemoradiotherapy (CRT) as the mainstay of treatment, with salvage surgery reserved for recurrent or residual disease [11, 12]. Long‐term outcomes following primary CRT are excellent, with a complete response rate of 70–80%, reduced local recurrence and better symptomatic control [13, 14, 15].

Several demographic and clinical features correlate with outcomes for ASCC patients. However, one feature which has not been explored is tumour morphology. ASCC morphology varies between exophytic, wart‐like lesions and ulcerated, flat, indurated lesions. Variations in outcomes according to morphology have been previously demonstrated in other malignancies such as gastric cancer and melanoma [16, 17]. Ulceration has been shown to be a negative prognostic factor, associated with advanced disease and aggressive tumour characteristics [18, 19]. This study aimed to investigate the effect of the morphology of ASCC on cancer outcomes.

## METHODS

### Design

This retrospective cohort study included all patients referred to Southeast Wales Anal Multidisciplinary Team (MDT) with suspected anal cancer between January 2003 and December 2022. Baseline characteristics including patient demographics were collected through the Welsh Clinical Portal. Patients were excluded if histology revealed non‐SCC tissue, if patients were lost to follow‐up or inadequate patient data was available.

Macroscopic tumour morphology was described at presentation by either a colorectal surgeon or the treating oncologist.

Exophytic tumours were defined as raised outgrowths of the tumour projecting from the epithelial surface. Ulceration was defined as the loss of intact epithelium overlying the tumour. Mixed tumours had a combination of these features.

### Outcomes

The primary outcome was overall survival (OS) at 1, 2 and 5 years.

Secondary outcomes included:

Complete response to treatment—defined as no residual disease within the radiotherapy field 3 months from treatment cessation.

Local recurrence rate—defined as ASCC presenting at the original site or surrounding structures, diagnosed either radiologically or histologically, after treatment at 1, 2 and 5 years.

Disease‐free survival—defined as the patient remaining free from ASCC at 1, 2 and 5 years.

Number of patients who underwent salvage surgery (APR or pelvic exenteration) for recurrent or treatment‐resistant disease.

### Statistical analysis

Relationships between patient characteristics and outcomes above were compared across the three morphology groups. Continuous patient demographic variables were compared between the three groups using the Mann–Whitney *U*‐test, and dichotomous variables were compared using the Chi‐squared test. Kaplan–Meier curves were produced to compare outcomes across each group. Univariable Cox proportional hazards regression was performed to assess associations between clinicopathological variables, including morphology and survival outcomes. Variables associated with a statistically significant result on univariable analysis were entered into a multivariable Cox regression model. Results are reported as hazard ratios (HRs) with 95% confidence intervals (CIs), and statistical significance was defined as *p* < 0.05. Statistics were performed using SPSS software Version 29, and all figures were generated using R Studio version 2024.09.1 +394.

## RESULTS

**FIGURE 1:**
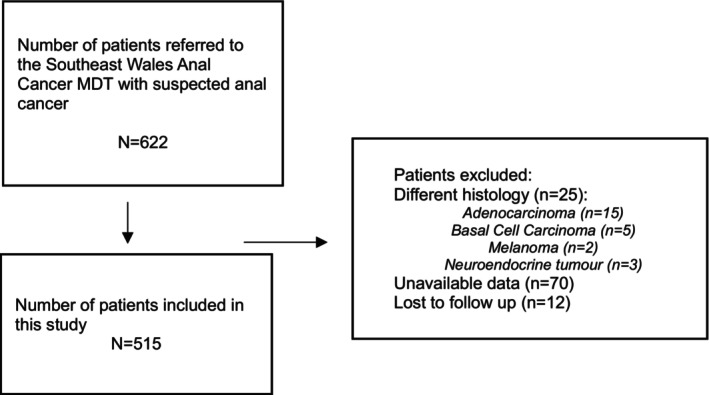
Flow chart representing the number of patients included in our study against the exclusion criteria.

There were 622 referrals with suspected ASCC to the Southeast Wales Anal MDT between January 2003 and December 2022. Of these, 107 patients were excluded, leaving 515 patients as summarised in Figure 1.

The female: male ratio was 340:175 (1.9:1) with a mean age of 63 years. There were 290 (56.3%) patients with exophytic morphology, 179 (34.8%) with ulcerated morphology and 46 (8.9%) with mixed morphology (Table 1).

**TABLE 1:** Presentation characteristics, risk factors and treatment modalities in anal squamous cell carcinoma.

	Exophytic (*n* = 290)	Ulcerated (*n* = 179)	Mixed (*n* = 46)	*p* Value
Age at diagnosis, years, mean (range)	62 (90–35)	65 (101–33)	62 (93–37)	0.232
Male, *n* (%)	92 (31.7%)	71 (39.7%)	12 (26.1%)	0.181
Female, *n* (%)	198 (68.3%)	108 (60.3%)	34 (73.9%)	0.105
Staging tumour, *n* (%)				
T1	71 (24.5%)	6 (3.4%)	4 (8.7%)	<0.001
T2	110 (37.9%)	58 (32.4%)	18 (39.1%)	0.435
T3	80 (27.6%)	60 (33.5%)	11 (23.9%)	0.273
T4	29 (10.0%)	55 (30.7%)	13 (28.3%)	<0.001
Staging nodal status, *n* (%)				
N0	179 (61.7%)	82 (45.8%)	25 (54.3%)	0.003
N1	61 (21.0%)	50 (27.9%)	14 (30.4%)	0.142
N2	50 (17.2%)	47 (26.3%)	7 (15.2%)	0.042
Staging metastasis, *n* (%)				
M0	284 (97.9%)	162 (90.5%)	45 (97.8%)	<0.001
M1	6 (2.1%)	17 (9.5%)	1 (2.2%)	<0.001
Smoking, *n* (%)				
Current smoker	93 (32.0%)	62 (34.6%)	20 (43.5%)	0.308
Ex‐smoker	84 (29.0%)	44 (24.6%)	15 (32.6%)	0.439
Never smoked	113 (39.0%)	73 (40.8%)	11 (23.9%)	0.103
History of head and neck malignancy, *n* (%)				
Yes	4 (1.4%)	3 (1.7%)	0 (0.0%)	0.681
No	286 (98.6%)	176 (98.3%)	46 (100%)	0.681
Anal intraepithelial neoplasia (AIN) surveillance, *n* (%)				
Yes	28 (9.7%)	2 (1.1%)	0 (0.0%)	<0.001
No	262 (90.3%)	177 (98.9%)	46 (100.0%)	<0.001
Human papilloma virus (HPV) status, *n* (%)				
Yes	65 (22.4%)	23 (12.8%)	8 (17.4%)	0.036
No	4 (1.4%)	3 (1.7%)	2 (4.3%)	0.360
Unknown	221 (76.2%)	153 (85.5%)	36 (78.3%)	0.052
Human immunodeficiency virus (HIV) status, *n* (%)				
Yes	14 (4.8%)	9 (5.0%)	2 (4.3%)	0.981
No	276 (96.2%)	170 (95.0%)	44 (95.7%)	0.943
Immunodeficiency, *n* (%)				
Yes	33 (11.4%)	18 (10.1%)	5 (10.9%)	0.905
No	257 (88.6%)	161 (89.9%)	41 (89.1%)	0.883
History of cervical/vaginal intraepithelial neoplasia (CIN/VIN) or vulval cancer, *n* (% of females)				
Yes	40 (20.2%)	9 (8.3%)	3 (8.8%)	0.006
No	168 (79.8%)	99 (91.7%)	31 (91.2%)	0.605
History of penile cancer, *n* (% of males)				
Yes	2 (2.2%)	0 (0.0%)	0 (0.0%)	0.459
No	90 (97.8%)	71 (100.0%)	12 (100.0%)	0.085
Men who have sex with men (MSM), *n* (% of males)				
Yes	7 (7.6%)	3 (4.2%)	2 (16.7%)	0.558
Unknown	85 (92.4%)	68 (95.8%)	10 (83.3%)	0.031
Treatment modality, *n* (%)				
Chemoradiotherapy	232 (80.0%)	133 (74.3%)	37 (80.4%)	0.322
Chemotherapy alone	0 (0.0%)	2 (1.1%)	0 (0.0%)	0.152
Radiotherapy alone	21 (7.2%)	12 (6.7%)	3 (6.5%)	0.967
Surgery (WLE)	30 (10.3%)	1 (0.6%)	2 (4.3%)	<0.001
Palliation	7 (2.4%)	31 (17.3%)	4 (8.7%)	<0.001

### Tumour size and stage

Patients with exophytic morphology had significantly smaller tumours at presentation, with 24.5% T1 compared to ulcerated (3.4%) and mixed (8.7%) groups (*p* < 0.001). Exophytic tumours also had significantly less nodal involvement with 61.7% N0, compared to 45.8% for ulcerated and 54.3% for mixed (*p* = 0.003) (Table 1).

Ulcerated and mixed morphology patients had significantly larger tumours at presentation with T4 rates of 10.0% in the exophytic group, versus 30.7% for ulcerated and 28.3% for mixed tumours (*p* < 0.001). The ulcerated group was more likely to have N2 nodal involvement at 26.3%, versus 17.2% for the exophytic and 15.2% for the mixed tumours (*p* = 0.042). Ulcerated morphology was associated with a greater risk of metastasis, with 9.5% presenting with metastatic disease, versus 2.1% in exophytic and 2.2% in mixed groups (*p* < 0.001).

### Risk factors

Data on known risk factors for the development and progression of ASCC were collected and summarised in Table 1.

For female patients, 52 (15.2%) had a history of CIN/VaIN/VIN or cancer. The exophytic group had a higher rate of previous gynaecological neoplasia at 20.2%, compared to 8.3% in the ulcerated group and 8.8% in the mixed tumours (*p* = 0.006).

Only 29 (5.6%) patients were already in an AIN surveillance programme. There were significantly more exophytic patients under such follow‐up at 9.7% compared to 1.1% of the ulcerated group and 0.0% of mixed tumours (*p* < 0.001).

Exophytic morphology was significantly associated with positive HPV status at 22%, compared to 12.8% for ulcerated and 17.4% for the mixed morphology group (*p* = 0.036). However, it should be noted that only 20% of patients had their HPV status recorded.

Only 25 (4.9%) patients were known to be HIV‐positive, with no significant difference between the three groups, but not all patients had a recorded test. Likewise, sexual history was not always recorded, and just 12/175 (6.8%) male patients were known to be MSM.

### Treatment

Four‐hundred two patients (78.1%) received CRT. Some patients who were deemed unfit for systemic chemotherapy received radiotherapy alone, with 36 patients (7.0%) having this treatment. For 33 patients (4.6%) with small, favourably sited tumours, primary surgery in the form of wide local excision was performed as first‐line treatment. Exophytic patients were more likely to receive this option (10.3%), compared to 0.6% of the ulcerated group and 4.3% of mixed tumours (*p* < 0.001). A significant minority of patients presented with advanced disease, and 42 (8.2%) were given palliative care as first line. These were significantly more in the ulcerated group (17.3% versus 2.4% of the exophytic group and 8.7% of mixed tumours) (*p* < 0.001) (Table 1).

### Overall survival

**FIGURE 2:**
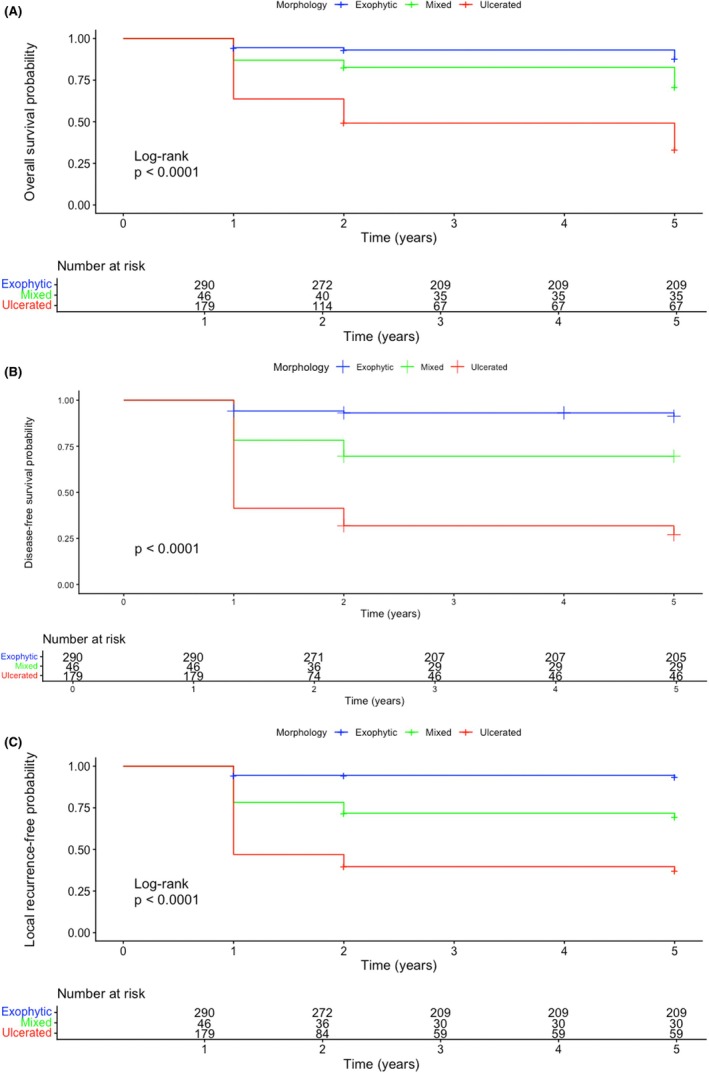
Kaplan–Meier curves for overall survival (OS), disease‐free survival (DFS) and local recurrence (LR) for anal squamous cell carcinoma patients across three different morphological subtypes. (A) OS LogRank *p* < 0.001 with associated number at risk table. (B) DFS LogRank *p* < 0.001 with associated number at risk table. (C) LR LogRank *p* < 0.001 with associated number at risk table.

Overall, 5‐year survival was 52.8%. Ulcerated morphology was associated with significantly fewer patients surviving 5 years—25.1% compared to 67.9% in the exophytic group and 65.2% in mixed tumours (*p* < 0.001) (Table 2). The Kaplan–Meier curve suggests a significantly higher survival probability for the exophytic patients (*p* < 0.001) (Figure 2A).

**TABLE 2:** Outcome data for overall survival, local recurrence, disease free survival and other secondary outcomes in anal squamous cell carcinoma.

	Exophytic	Ulcerating	Mixed	*p* Value
OS at 5 years	197 (67.9%)	45 (25.1%)	30 (65.2%)	<0.001
os at 2 years	268 (92.4%)	87 (48.6%)	38 (82.6%)	<0.001
OS at 1 year	273 (94.1%)	112 (62.6%)	40 (87.0%)	<0.001
LR at 5 years	18 (6.2%)	112 (62.6%)	14 (30.4%)	<0.001
LR at 2 years	16 (5.5%)	108 (60.3%)	13 (28.2%)	<0.001
LR at 1 year	16 (5.5%)	96 (53.6%)	10 (21.7%)	<0.001
DFS at 5 years	203 (70.0%)	39 (21.8%)	29 (63.0%)	<0.001
DFS at 2 years	268 (92.4%)	57 (31.8%)	32 (69.6%)	<0.001
DFS at 1 year	272 (93.8%)	74 (41.3%)	36 (78.3%)	<0.001
Salvage surgery rate (% of local recurrence at 5 years)	5 (27.8%)	12 (10.7%)	2 (14.3%)	0.059
Metastatic disease	6 (2.1%)	17 (9.5%)	1 (2.2%)	<0.001
Complete response to treatment	278 (95.9%)	82 (45.8%)	38 (82.6%)	<0.001

### Disease‐free survival

There were 271 patients (52.6%) with DFS at 5 years. A similar trend across the groups was seen with DFS as with OS. Ulcerated morphology was associated with significantly worse DFS across all timepoints, with 5‐year DFS of 21.8% compared to 70.0% for exophytic tumours and 63.0% in the mixed group (*p* < 0.001) (Figure 2B).

### Local recurrence

**FIGURE 3:**
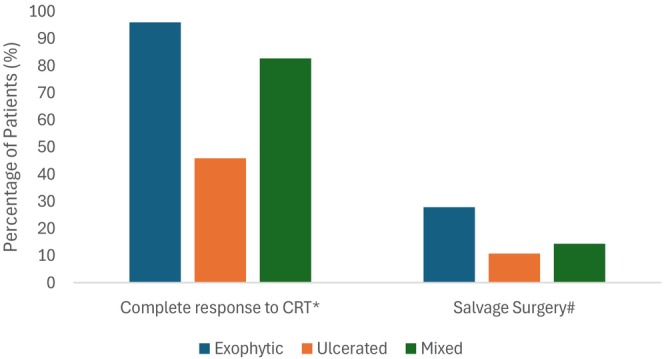
Bar graph depicting other secondary outcomes between different morphological subtypes of anal squamous cell carcinoma. Complete response to CRT (*p < 0.001) and salvage surgery rate expressed as percentage of patients with local recurrence at 5 years (^#^ *p* = 0.059).

One hundred forty‐four patients (28.0%) had local recurrence (LR) within 5 years. The ulcerating morphology group had significantly more recurrences across all timepoints at 62.6%, versus 6.2% in the exophytic group and 30.4% for the mixed group (*p* < 0.001) (Figure 2C). Patients who had LR were assessed to determine if they were suitable for salvage surgery. In total, 19 patients underwent salvage surgery after LR. Of these, 12 patients had ulcerated morphology, but no significant difference was observed in the number of salvage procedures between the three groups (*p* = 0.059) due to the small total number of patients (Figure 3).

A univariable analysis using a time‐to‐event Cox regression model was performed to analyse the independent effects of different clinical variables, including morphology, on clinical outcomes (Table 3). Poor 5‐year overall survival was seen statistically significantly with increasing age, male patients, greater tumour stage, greater nodal stage, presence of metastasis and morphology. Ulcerated and mixed morphology were associated with significantly higher hazard of death compared to exophytic morphology—HR 7.391 (4.979–10.972) *p* < 0.001 and HR 2.557 (1.342–4.872) *p* < 0.001, respectively. Having chemotherapy alone and palliation compared to chemoradiotherapy was also associated with markedly higher hazard ratios. Interestingly, having CIN/VIN/Vulval Ca and surgery compared to chemoradiotherapy was associated with better survival.

**TABLE 3:** Univariable and multivariable Cox regression analysis of 5‐year overall survival, 5‐year disease‐free survival and local recurrence.

	Overall survival 5 years‐univariable analysis		Overall survival 5 years‐multivariable analysis	
	Hazard ratio (95%CI)	*p* Value	Hazard ratio (95%CI)	*p* Value
Age at diagnosis	1.032 (1.020–1.045)	<0.001	1.021 (1.007–1.035)	0.003
Male vs. Female	1.556 (1.144–2.145)	0.005	1.401 (0.989–1.986)	0.058
Currently smoking vs. Non‐smoker	0.820 (0.573–1.173)	0.276		
Ex smoker vs. Non‐smoker	0.685 (0.457–1.024)	0.065		
Tumour stage (T)	—	<0.001	—	0.011
T4 vs. T1	12.567 (4.518–34.956)	<0.001	5.968 (1.720–20.707)	0.005
T3 vs. T1	9.971 (3.628–27.403)	<0.001	5.292 (1.560–17.950)	0.008
T2 vs. T1	5.192 (1.867–14.436)	0.002	3.525 (1.049–11.847)	0.042
Nodal stage (N)	—	0.006	—	0.301
N2 vs. N0	1.800 (1.238–2.617)	0.002	1.340 (0.868–2.067)	0.187
N1 vs. N0	1.474 (1.004–2.164)	0.048	0.983 (0.635–1.520)	0.937
Metastasis M1 vs. M0	3.143 (1.866–5.293)	<0.001	0.993 (0.531–1.855)	0.981
Morphology	—	<0.001	—	<0.001
Ulcerated vs. Exophytic morphology	7.391 (4.979–10.972)	<0.001	5.160 (3.338–7.979)	<0.001
Mixed vs. Exophytic morphology	2.557 (1.342–4.872)	0.004	2.299 (1.179–4.482)	0.015
AIN surveillance	0.456 (0.187–1.111)	0.084		
HPV positivity	0.856 (0.198–3.707)	0.836		
HIV positivity	1.167 (0.776–1.755)	0.458		
Immunocompromised	1.198 (0.749–1.914)	0.451		
CIN/VIN/Vulval cancer	0.385 (0.180–821)	0.014	0.944 (0.424–2.100)	0.887
Head and neck cancer	1.034 (0.515–2.076)	0.926		
Surgery vs. Chemoradiotherapy	0.211 (0.052–0.857)	0.030	1.060 (0.223–5.042)	0.942
Radiotherapy alone vs. Chemoradiotherapy	1.734 (0.991–3.034)	0.054	1.359 (0.751–2.459)	0.311
Chemotherapy alone vs. Chemoradiotherapy	6.893 (1.691–28.107)	0.007	3.058 (0.626–14.950)	0.167
Palliation vs. Chemoradiotherapy	6.801 (4.623–10.005)	<0.001	2.948 (1.889–4.599)	<0.001

	Disease free survival 5 years‐ univariable analysis		Disease free survival 5 years‐ multivariable analysis	
	Hazard ratio (95%CI)	*p* Value	Hazard ratio (95%CI)	*p* Value
Age at diagnosis	1.024 (1.012–1.037)	<0.001	1.014 (1.001–1.026)	0.034
Male vs. Female	1.330 (0.976–1.812)	0.071		
Currently smoking vs. Non‐smoker	0.908 (0.639–1.290)	0.590		
Ex smoker vs. Non‐smoker	0.799 (0.543–1.176)	0.255		
Tumour stage (T)	—	<0.001	—	0.172
T4 vs. T1	8.918 (3.831–20.760)	<0.001	1.855 (0.707–4.868)	0.210
T3 vs. T1	6.231 (2.696–14.403)	<0.001	1.580 (0.609–4.100)	0.347
T2 vs. T1	3.338 (1.422–7.834)	0.006	1.153 (0.449–2.960)	0.767
Nodal stage (N)	—	<0.001	—	0.502
N2 vs. N0	1.913 (1.318–2.776)	<0.001	1.257 (0.827–1.910)	0.284
N1 vs. N0	1.798 (1.252–2.582)	0.001	1.220 (0.814–1.829)	0.335
Metastasis M1 vs. M0	3.198 (1.975–5.177)	<0.001	1.058 (0.601–1.865)	0.844
Morphology	—	<0.001	—	<0.001
Ulcerated vs. Exophytic morphology	10.855 (6.990–16.856)	<0.001	7.698 (4.840–12.242)	<0.001
Mixed vs. Exophytic morphology	3.866 (2.000–7.474)	<0.001	3.144 (1.608–6.145)	<0.001
AIN surveillance	0.089 (0.012–632)	0.016	0.394 (0.050–3.083)	0.375
HPV positivity	0.915 (0.213–3.928)	0.905		
HIV positivity	0.709 (0.314–1.602)	0.409		
Immunocompromised	0.994 (0.610–1.622)	0.982		
CIN/VIN/Vulval cancer	0.429 (0.211–0.873)	0.020	0.927 (0.448–1.918)	0.838
Head and neck cancer	0.897 (0.222–3.616)	0.878		
Surgery vs. Chemoradiotherapy	0.200 (0.049–0.809)	0.024	0.664 (0.150–2.935)	0.589
Radiotherapy alone vs. Chemoradiotherapy	1.003 (0.525–1.916)	0.992	0.885 (0.455–1.724)	0.720
Chemotherapy alone vs. Chemoradiotherapy	4.968 (1.222–20.193)	0.025	1.803 (0.394–8.249)	0.447
Palliation vs. Chemoradiotherapy	4.788 (3.299–6.947)	<0.001	2.118 (1.369–3.277)	<0.001

	Local recurrence 5 years‐ univariable analysis		Local recurrence 5 years‐ multivariable analysis	
	Hazard ratio (95%CI)	*p* Value	Hazard ratio (95%CI)	*p* Value
Age at diagnosis	1.030 (1.017–1.043)	<0.001	1.014 (1.001–1.028)	0.039
Male vs. Female	1.451 (1.043–2.020)	0.027	1.337 (0.939–1.903)	0.107
Currently smoking vs. Non‐smoker	0.902 (0.616–1.320)	0.596		
Ex smoker vs. Non‐smoker	0.853 (0.566–1.287)	0.449		
Tumour stage (T)	—	<0.001	—	0.163
T4 vs. T1	10.687 (3.844–29.711)	<0.001	2.171 (0.694–6.787)	0.183
T3 vs. T1	8.185 (2.969–22.564)	<0.001	1.974 (0.639–6.104)	0.238
T2 vs. T1	4.151 (1.479–11.646)	0.007	1.334 (0.435–4.087)	0.614
Nodal stage (N)	—	0.006	—	0.889
N2 vs. N0	1.732 (1.156–2.594)	0.008	1.096 (0.697–1.725)	0.691
N1 vs. N0	1.702 (1.156–2.507)	0.007	1.102 (0.717–1.692)	0.659
Metastasis M1 vs. M0	2.834 (1.683–4.773)	<0.001	1.036 (0.566–1.897)	0.909
Morphology	—	<0.001	—	<0.001
Ulcerated vs. Exophytic morphology	11.548 (7.003–19.042)	<0.001	7.482 (4.425–12.651)	<0.001
Mixed vs. Exophytic morphology	5.084 (2.528–10.223)	<0.001	4.126 (2.025–8.408)	<0.001
AIN surveillance	0.106 (0.015–0.759)	0.025	0.615 (0.077–4.910)	0.646
HPV positivity	0.864 (0.201–3.726)	0.845		
HIV positivity	0.837 (0.369–1.895)	0.669		
Immunocompromised	1.098 (0.662–1.822)	0.717		
CIN/VIN/Vulval cancer	0.374 (0.165–0.848)	0.018	0.907 (0.387–2.126)	0.823
Head and neck cancer	1.043 (0.258–4.210)	0.953		
Surgery vs. Chemoradiotherapy	0.126 (0.018–0.906)	0.040	0.407 (0.053–3.159)	0.390
Radiotherapy alone vs. Chemoradiotherapy	1.243 (0.647–2.389)	0.514	1.084 (0.553–2.125)	0.813
Chemotherapy alone vs. Chemoradiotherapy	2.381 (0.332–17.089)	0.388	0.998 (0.126–7.898)	0.999
Palliation vs. Chemoradiotherapy	5.485 (3.746–8.032)	<0.001	2.470 (1.581–3.857)	<0.001

*Note*: Factors found significant in univariable analysis are added to form a multivariable analysis. Those found non‐significant in univariable analysis are marked in grey in multivariable. All effects are expressed as Hazard Ratios (HR) and 95% Confidence Intervals (CI). *p* < 0.05 is considered statistically significant.

On cox regression model multivariable analysis, age at diagnosis [HR 1.021 (1.007–1.035) *p* = 0.003], tumour staging across all compared to T1 (*p* = 0.011), palliation compared to chemoradiotherapy [HR 2.948 (1.889–4.599) *p* < 0.001] and ulcerated [HR 5.160 (3.338–7.979) *p* < 0.001] and mixed morphology [HR 2.299 (1.179–4.482) *p* = 0.015] compared to exophytic was associated with significantly worse overall survival.

For DFS, ulcerated and mixed morphology were significantly associated with worse survival in univariable [HR 10.855 (6.990–16.856) *p* < 0.001 and HR 3.866 (2.000–7.474) *p* < 0.001, respectively] and multivariable analysis [HR 7.698 (4.840–12.242) *p* < 0.001 and HR 3.144 (1.608–6.145) *p* < 0.001, respectively]. For local recurrence, as well, ulcerated and mixed morphology were significantly associated with greater local recurrence in univariable [HR 11.548 (7.003–19.042) *p* < 0.001 and HR 5.084 (2.528–10.223) *p* < 0.001 respectively] and in multivariable analysis [HR 7.482 (4.425–12.651) *p* < 0.001 and HR 4.126 (2.025–8.408) *p* < 0.001 respectively]. All other factors and corresponding analyses can be seen in Table 3.

Considering the potential confounding effects of palliative treatment intent (which was significant in multivariable analysis) across all outcome measures and ulcerated morphology had the greatest proportion of patients with this treatment intent, a subgroup analysis of outcomes was performed with patients treated with curative intent to assess if the association of morphology and clinical outcomes persisted. All patients who were palliated were excluded (*n* = 42).

This is shown in Table 4. Across all outcome measures, ulcerated morphology had significantly worse 5‐year OS (30.4% vs. 69.6% vs. 69.0%, *p* < 0.001), worse 5‐year DFS (26.4% vs. 71.4% vs. 69.0%, *p* < 0.001) and greater 5‐year local recurrence rate (54.7% vs. 4.2% vs. 23.8%, *p* < 0.001).

**TABLE 4:** Sub‐group analysis of overall survival at 5 years, disease‐free survival at 5 years and local recurrence in patients treated with only curative intent, excluding those treated with palliative intent across different morphology groups.

	Exophytic (*n* = 283)	Ulcerated (*n* = 148)	Mixed (*n* = 42)	*p* Value
OS at 5 years	197 (69.6%)	45 (30.4%)	29 (69.0%)	<0.001
OS at 2 years	267 (94.3%)	86 (58.1%)	37 (88.1%)	<0.001
OS at 1 year	271 (95.8%)	105 (70.9%)	39 (92.9%)	<0.001
DFS at 5 years	202 (71.4%)	39 (26.4%)	29 (69.0%)	<0.001
DFS at 2 years	267 (94.3%)	57 (38.5%)	32 (76.2%)	<0.001
DFS at 1 year	271 (95.8%)	74 (50.0%)	36 (85.7%)	<0.001
Local recurrence at 5 years	12 (4.2%)	81 (54.7%)	10 (23.8%)	<0.001
Local recurrence at 2 years	10 (3.5%)	77 (52.0%)	9 (21.4%)	<0.001
Local recurrence at 1 year	10 (3.5%)	65 (43.9%)	6 (14.3%)	<0.001

## DISCUSSION

This series reports 515 consecutive ASCC patients, treated by an anal cancer MDT over the past 20 years, representing the combined outcomes of surgical and oncological management. Given the long duration of this retrospective series, optimum management of ASCC has evolved, but the MDT endeavoured to keep up with developments and appropriate clinical trials to deliver state‐of‐the‐art care. This is the first report to demonstrate a correlation between the morphology of ASCC and oncological outcomes.

At presentation, ulcerated morphology was associated with significantly more advanced disease—significantly larger tumour sizes, increased nodal involvement and metastatic disease. Ulceration has previously been demonstrated as a negative prognostic factor in melanomas and gastric cancer, with guidelines accommodating for a greater stage of disease due to the presence of ulceration [16, 20, 21, 22]. In melanomas, ulceration is associated with increased mitotic rate, greater vascular invasion and increased inflammatory response with increased number of pro‐inflammatory cytokines correlating with aggressive cancer biology [23, 24]. This parallels the findings of this study linking ulcerated ASCC morphology with poor oncological outcomes. It could be argued that all anal cancers initially present as exophytic lesions and that the more aggressive ones progress to an ulcerative stage, explaining the mixed morphology observed as a transition phase. However, it may be possible that ulcerated ASCC can arise de novo and exist as a separate entity. This warrants further investigation into the development and progression of these lesions and how they compare at a histological level.

While ulcerated morphology remained associated with poorer outcomes after multivariable adjustment, these findings should be interpreted cautiously. The observed survival differences should not be attributed solely to intrinsic tumour biology, as morphology groups differed substantially in baseline prognostic characteristics. In particular, ulcerated tumours were associated with more advanced stage at presentation, greater metastatic burden, lower surveillance exposure and higher rates of palliative treatment allocation. These factors may reflect later disease detection and greater baseline disease burden, which could partly mediate the poorer outcomes observed in the ulcerated group. Therefore, although ulcerated morphology may represent a clinically aggressive phenotype, its prognostic significance may arise from a combination of biological behaviour, delayed presentation, disease stage and treatment intent. Residual confounding cannot be excluded, and these findings should be validated in larger cohorts with detailed adjustment for diagnostic pathway, stage and treatment allocation.

The exophytic group had a significantly greater HPV‐positivity rate at 22.4%, compared to 12.8% for ulcerated and 17.4% for mixed tumours (*p* = 0.036). One possible explanation is that HPV causes proliferative epithelial changes producing wart‐like exophytic lesions [25]. These patients may therefore present with “warts” to genitourinary clinics where HPV testing is more likely to be offered. Even though HPV is associated with 70–90% of cases of ASCC [26], our cohort only had an HPV‐positivity rate of 18.6% due to limited HPV‐testing, especially during the early years of this series. In this study, the exophytic group had a significantly higher incidence of cervical neoplasia, which correlates with the higher incidence of HPV‐positivity in this group.

Only 30/515 (5.8%) patients in this series were already on the AIN surveillance programme at the time of diagnosis of ASCC, because no such program was available during the early years of this study. The risk of transformation from AIN to ASCC has been the subject of much research and varies from 11% to 32% depending on multiple factors, including the presence of multifocal disease, HIV status and grade of AIN [27, 28].

CRT is the current gold standard for treatment of ASCC and is far superior to radiotherapy alone [11]. This cohort is representative, with 78.1% of patients receiving CRT as first‐line treatment. Radiotherapy alone was given to 7% of patients, mainly because of frailty or co‐morbidity. Complete response occurred in 77.3% of patients, in line with rates of 70–80% in the literature [14, 15, 29]. Exophytic patients had a significantly better response to CRT at 95.9%, with ulcerated patients having the worst complete response rate at 45.8% and mixed morphology tumours having an intermediate response rate of 82.6%.

Primary palliative treatment was reserved for those with advanced (incurable) disease and was more common in the ulcerated group. Since palliative treatment was significantly associated with worse survival in multivariable analysis, a subgroup analysis was performed in patients managed with curative intent. This was undertaken because ulcerated tumours were more frequently associated with palliative treatment allocation, raising the possibility that outcome differences in the overall cohort may partly reflect advanced disease burden and treatment intent rather than morphology alone. However, even in this cohort of patients treated with curative intent, ulcerated morphology was associated with significantly worse survival and greater recurrence rates. Nevertheless, these findings should be interpreted as supportive rather than definitive evidence of an independent prognostic role for morphology.

Multivariable Cox regression was performed to assess whether tumour morphology remained associated with outcome after adjustment for key clinicopathological and treatment‐related factors. In this analysis, morphology remained independently associated with 5‐year overall survival, with ulcerated and mixed tumours demonstrating significantly poorer outcomes compared with exophytic tumours. This suggests that macroscopic morphology may provide additional prognostic information beyond conventional staging alone. However, the persistence of some variables after adjustment, including tumour stage in OS and DFS, as well as palliative intent across all three domains, highlights the complex interaction between morphology, disease burden and treatment allocation.

Most (84.7%) LR occurred within the first year post‐treatment, which could be because of increased surveillance during this period or because ulcerated tumours are more biologically aggressive and manifest with residual/recurrent disease more quickly.

Patients with residual or recurrent ASCC were considered for salvage surgery. Despite having more LR, the ulcerated group had a lower percentage of patients undergoing salvage surgery. The biological reasons for this are unknown, but may be because ulcerated patients have more advanced tumour presentation, which is biologically less sensitive to CRT. Some patients are too frail to have extensive surgery or cope with at least one stoma.

This is a retrospective, single‐centre study of a relatively rare condition and therefore conclusions should be drawn with caution. The definition of macroscopic morphology relies on the opinion of the examining clinician, which, as there is no standard classification of ASCC morphology, may cause discrepancies in reporting. It will be important to elucidate whether exophytic and ulcerated are two distinct biological entities, or whether all ASCCs begin as exophytic tumours, but the more aggressive ones ulcerate.

Additional baseline imbalances between morphology groups may also have influenced outcome differences. Patients with exophytic tumours had higher rates of AIN surveillance, HPV positivity and prior CIN/VIN history, which may have increased the likelihood of earlier detection and contributed to more favourable outcomes. Although AIN surveillance, HPV status and prior CIN/VIN history differed between morphology groups, these variables were not significantly associated with survival on univariable or multivariable analysis. Therefore, they are unlikely to independently explain the observed survival differences. However, they may still have influenced the pathway to diagnosis, with patients under surveillance or with prior HPV‐associated disease potentially being diagnosed at an earlier stage. As such, the prognostic association observed for morphology may reflect a combination of tumour biology, stage at presentation and differences in detection pathways. These findings should therefore be interpreted with caution, and residual confounding by surveillance intensity and diagnostic pathway cannot be excluded. This should prompt the need for larger prospective studies with propensity score matching to better explore the oncological effects of morphology.

## CONCLUSION

This is the first study to show that ASSC morphology significantly predicts survival and response to treatment. Ulcerated morphology presents with more advanced disease, and these patients have a poor response to chemoradiotherapy, resulting in a higher incidence of residual or recurrent disease. Ulcerated morphology is also a significant risk factor for both OS and DFS at 1, 2 and 5 years.

## AUTHOR CONTRIBUTIONS

**Thomas Rackley:** Writing – review and editing; project administration; resources; supervision; data curation. **James Horwood:** Conceptualization; writing – review and editing; project administration; data curation; supervision; resources. **Rachel Hargest:** Conceptualization; writing – original draft; writing – review and editing; project administration; data curation; supervision; resources. **Alwin Puthiyakunnel Saji:** Conceptualization; writing – original draft; methodology; validation; visualization; writing – review and editing; software; formal analysis; project administration; data curation; investigation.

## FUNDING INFORMATION

There are no funding sources for this paper.

## CONFLICT OF INTEREST STATEMENT

The authors declare no conflicts of interest.

## ETHICS STATEMENT

Due to the retrospective nature of this study, the use of non‐identifiable patient data and the absence of any experimental intervention other than that of standard of care, ethical approval from an ethics committee was not required. The project was, however, registered with the Clinical Audit department at the Velindre Cancer Centre.

## CONSENT

Consent from patients is not required in the absence of any intervention, experiment or departure from standard clinical practice.

## Data Availability

The data that support the findings of this study are available on request from the corresponding author. The data are not publicly available due to privacy or ethical restrictions.
